# The Effect of Volume-Flow Discordance on Survival in Severe Aortic Stenosis

**DOI:** 10.1016/j.jacasi.2025.07.016

**Published:** 2025-08-27

**Authors:** Sara L. Hungerford, Ning Song, Brandon Loo, Eleanor Rye, Hari Sritharan, Kay D. Everett, Christopher S. Hayward, Navin K. Kapur, David W.M. Muller, Audrey I. Adji

**Affiliations:** a The CardioVascular Center Tufts, Boston, Massachusetts, USA; b Faculty of Medicine and Health, University of Sydney, Sydney Australia; c Department of Cardiology, Royal North Shore Hospital, Sydney, Australia; d Faculty of Health and Medicine the University of New South Wales, Sydney, Australia; e Department of Cardiology, St Vincent’s Hospital, Sydney, Australia; f Victor Chang Cardiac Research Institute, Sydney, Australia

**Keywords:** aortic stenosis, echocardiography, flow rate, left ventricular function, stroke volume indexed, volume-flow discordance

## Abstract

**Background:**

Current flow (Q) assessment in aortic stenosis (AS) uses stroke volume index (SVi), a volume (V)–based measure. However, V differs fundamentally from Q, which is defined as volume per unit time (mL/s).

**Objectives:**

This study evaluates the prognostic significance of volume-flow (V-Q) discordance in patients with severe AS (aortic valve area <1 cm^2^) undergoing transcatheter aortic valve replacement (TAVR).

**Methods:**

We studied 291 patients >65 years of age who underwent TAVR over 5 years (median follow-up, 3.0 years; [Q1-Q3: 3.0-3.0 years]). Aortic flow was assessed using Doppler echocardiography; transaortic flow rate (TFR) was calculated mathematically. Low V-Q discordance was defined as SVi <35 mL/m^2^ with TFR >210 mL/s; normal V-Q discordance as SVi >35 mL/m^2^ with TFR <210 mL/s.

**Results:**

V-Q discordance was observed in 29% of patients (15% low, 14% normal). Among those with SVi <35 mL/m^2^, discordance was more frequent in patients without hypertension (75% vs 65%), coronary disease (57% vs 35%), or diabetes (15% vs 2%; all *P* < 0.05). Diastolic blood pressure was lower (mean SD: 66+15 vs 59+14 mm Hg; *P* = 0.018), and arterial compliance was higher (median and Q1-Q3: 1.3 [1.1-1.6] vs 1.0 [0.9-1.2] mL/mm Hg; *P* = 0.028), independent of SVi and left ventricular ejection fraction (both *P* > 0.05). Low V-Q discordance was associated with improved 3-year survival (86.0% [95% CI: 72.3%-95.1%] vs 73.8% [95% CI: 64.3%-82.1%]; log-rank *P* = 0.030) and was a stronger survival predictor (Akaike information criterion [AIC]: 23.79; *P* = 0.013) than SVi <35 mL/m^2^ (AIC: 29.59; *P* = 0.047) or TFR <210 mL/s (AIC: 32.80; *P* = 0.049).

**Conclusions:**

V-Q discordance occurs in nearly one-third of patients with AS post-TAVR and offers superior prognostic value over existing SVi and TFR thresholds.

Recognition of the importance of flow (Q) in the assessment of aortic stenosis (AS), particularly in those with “discordant” indices of severity, has gained momentum in recent years. Current assessment of Q in AS uses a volume (V)–based approach and stroke volume index (SVi). In reality, V is fundamentally different from Q, with the latter defined as volume per unit time (mL/s).

Recent work has highlighted the value of assessing transaortic flow rate (TFR) as an alternative to SVi in certain AS populations.^1^^,^^2^ A TFR threshold of ≤210 mL/s has been widely adopted, having been associated with increased cardiovascular and all-cause mortality across the spectrum of AS severity, from mild to severe disease.^3^, ^4^, ^5^, ^6^ More recently, Springhetti et al^7^ proposed a slightly higher cutoff of <218 mL/s to improve outcome prediction in moderate AS. Beyond prognostication, TFR also offers important hemodynamic insights into properties of the systemic circulation in AS, independent of aortic valve area (AVA), and left ventricular ejection fraction (LVEF).^4^^,^^8^, ^9^, ^10^

Limited prior studies have explored the interplay of TFR with SVi. Namasivayam et al^1^ and Sen et al^2^ found that nearly one-third of AS patients exhibited V-Q discordance, and that discordance—particularly when characterized by low TFR in the context of preserved SVi—was associated with adverse outcomes. Further, Vamvakidou et al^11^ showed that in patients with low SVi, those with concordantly low TFR had worse outcomes compared to those with discordantly preserved TFR. These prior studies included very few patients with discordant severe AS (ie, does not meet the criteria for severe AS across all parameters) undergoing transcatheter aortic valve replacement (TAVR), where increased systemic vascular afterload may reasonably be expected to exert an undue effect AS hemodynamics.^12^

Collectively, growing clinical momentum supporting the use of TFR in the assessment of AS in patients undergoing TAVR creates impetus to better understand the effect of V-Q discordance. Theoretically, elevated blood pressure (BP), increased systemic vascular afterload, and comorbidities that contribute to arteriosclerosis or aortic stiffening may reduce TFR—independent of SVi—and result in poorer clinical outcomes.^13^^,^^14^

The objective of this study was to determine whether V-Q discordance was associated with survival outcomes in patients with severe AS undergoing TAVR. We hypothesized that low V-Q discordance would: 1) be linked to improved survival in patients with “low-flow” AS (ie, SVi <35 mL/m^2^ but TFR ≥210 mL/s); and 2) enhance mortality prediction beyond the use of a single SVi (the current guideline standard) or TFR threshold alone.^15^^,^^16^

## Methods

This was a registry analysis of 291 elderly patients ≥65 years of age with discordant symptomatic or severe AS who underwent TAVR over a 3-year period. The study protocol conformed to ethical guidelines of the 1975 Declaration of Helsinki, was approved by the local institutional review board (LNR/17/SVH/195), and all subjects gave informed consent. Data supporting the findings of this study are available from the corresponding author upon reasonable request.

### Hemodynamic study methods

**Figure 1:**
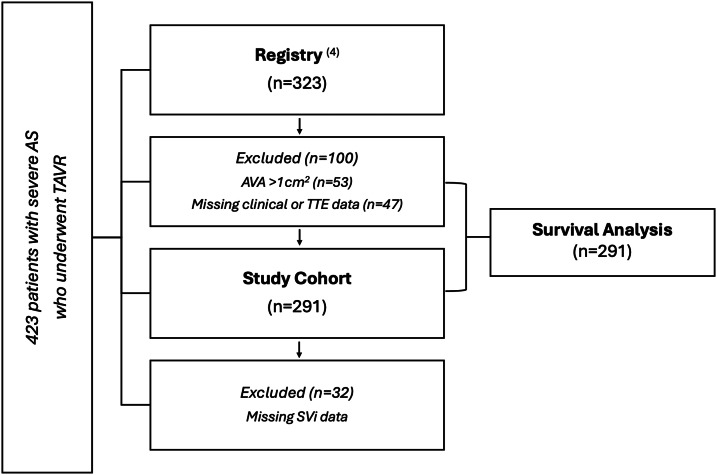
Study Cohort Selection Flow Diagram 423 patients >65 years of age with severe aortic stenosis (AS) underwent transcatheter aortic valve replacement (TAVR) over a 5-year period.^4^ Fifty-three patients were excluded due to aortic valve area (AVA) >1 cm^2^, and 47 due to incomplete clinical or echocardiographic data. A further 32 patients were excluded due to missing stroke volume index (SVi) data at time of transthoracic echocardiogram (TTE), leaving 291 patients with complete data available for 3-year survival analysis.

Inclusion criteria for the study were as follows: ≥65 years of age; AVA ≤ 1cm^2^; NYHA functional class ≥II, 2-dimensional transthoracic echocardiogram (TTE) and BP recording with SVi and TFR measurements available before TAVR as specified previously.^4^ Exclusion criteria included patients <65 years of age, missing clinical or TTE data, bicuspid aortic valve, and those with more than mild aortic or mitral regurgitation (either before or after TAVR).^4^ A total of 423 registry patients were screened. Of these, 53 were excluded due to an AVA >1 cm^2^, and 47 due to incomplete clinical or TTE data. A further 32 patients were excluded due to missing SVi measurements at the time of TTE acquisition, leaving 291 patients with complete data for survival analysis. All included patients had follow-up clinical outcome data available 3-years post-TAVR as per registry inclusion criteria.^4^ The primary endpoint was all-cause mortality; deaths were identified via a review of patient records or by contacting relatives (Figure 1).^4^

TTE images were acquired using an EPIQ 7 ultrasound machine (Philips Healthcare). LVEF was assessed using Simpson’s biplane method and considered impaired if ≤50%. AS severity was quantified following American Society of Echocardiography guidelines.^4^^,^^16^^,^^17^ Left ventricular outflow tract diameter was measured from the parasternal long-axis view in mid-systole, using the inner edge–to–inner edge convention, 0.5 to 1.0 cm below the aortic annulus.^16^ Left ventricular outflow tract velocity time integral was assessed using pulsed-wave Doppler from the apical 5-chamber view, with alignment of the Doppler beam parallel to subvalvular flow and placement of the sample volume just proximal to the aortic valve.^16^^,^^17^ The mean gradient was derived from the aortic valve velocity using continuous-wave Doppler, applying the modified Bernoulli equation, and reported as the average after excluding the highest and lowest values. The AVA was calculated using the continuity equation and expressed as the average of 5 measurements, excluding highest and lowest values.^4^^,^^16^^,^^17^ The TFR was measured using a mathematical derivation method to minimise colinearity^1^^,^^4^

TFR=AVA×Vm

where *AVA* (cm^2^) is AVA and *Vm* (m/s) is mean transvalvular velocity. This approach is based on the principle that TFR is not only the ratio of stroke volume to ejection time but also the product of AVA and mean velocity.^1^^,^^4^

As per AS reporting guidelines, classical severe AS was defined as a mean gradient ≥40 mm Hg, peak jet velocity ≥4.0 m/s, and an AVA ≤1 cm^2^ (or indexed ≤0.6 cm^2^/m^2^). “Discordant” severe AS referred to patients with moderate AS by peak velocity/gradient criteria (ie, 3.0 to 3.9 m/s and 20 to 39 mm Hg, respectively) but severe AS by AVA criteria (AVA ≤1.0 cm^2^). True severe low-flow, low-gradient (LF-LG) AS was diagnosed in patients with an AVA ≤1 cm^2^, LVEF <50%, mean gradient <40 mm Hg, and an SVi ≤35 mL/m^2^ at rest who were confirmed as having severe by dobutamine stress echocardiography (ie, AVA remained ≤1 cm^2^ with a peak gradient increase to ≥40 mm Hg during pharmacologic stress). Patients with pseudo-severe LF-LG AS and/or who underwent adjuvant computed tomography calcium scoring when dobutamine stress echocardiography was inconclusive or not feasible, were excluded. Patients with paradoxical LF-LG AS had an AVA ≤1 cm^2^, LVEF ≥50%, mean gradient <40 mm Hg, and an SVi ≤35 mL/m^2^.^4^^,^^16^^,^^17^ Echocardiographic measurements were manually obtained through image review by study investigators. For patients in atrial fibrillation at the time of study, all Doppler measurements were averaged over 5 consecutive cardiac cycles to reduce beat-to-beat variability. Premature or post-ectopic beats were excluded from analysis. All measurements were independently reviewed and verified by a second investigator to ensure accuracy and consistency. Values were not extracted from automated reports.

Four hemodynamic groups were identified based on SVi and TFR parameters. Group 1, classified as low V-Q concordance, had an SVi ≤35 mL/m^2^ and a TFR <210 mL/s. Group 2, defined as low V-Q discordance, had an SVi ≤35 mL/m^2^ but paradoxically a TFR ≥210 mL/s. Group 3, categorized as normal V-Q concordance, had an SVi >35 mL/m^2^ and a TFR ≥210 mL/s. Finally, Group 4, defined as normal V-Q discordance, had an SVi >35 mL/m^2^ but a TFR <210 mL/s.

### Statistical analysis

A dichotomous endpoint, two independent sample study design with an alpha of 0.05, beta of 0.2, and power of 0.8 (estimated V-Q discordance incidence of 30%) determined a minimum sample size of 196 patients for registry analysis. Normally distributed variables are reported as mean ± SD. Non-normally distributed variables are reported as median (Q1-Q3). Baseline characteristics were compared using the Student’s *t*-test for normally distributed continuous variables, the Mann–Whitney *U* test for non-normally distributed continuous variables, and the chi square or Fisher exact test for categorical variables, unless otherwise specified.

Survival analyses were performed using both univariable and multivariable Cox proportional hazards regression and logistic regression models. Cox models were used to evaluate time-to-event outcomes, with age, sex, LVEF, AVA, aortic valve velocity, and AS category included as covariates, reflecting known prognostic factors while accounting for events-per-variable constraints. The proportional hazards assumption was formally assessed using Schoenfeld residuals via the cox.zph() function in R. Both global and individual *P* values were reviewed, and no significant violations were detected (global *P* = 0.071), supporting model validity. Visual inspection of log-minus-log survival plots further confirmed the assumption.

Model performance was evaluated using likelihood ratio testing, Akaike information criterion (AIC), and Harrell’s C-statistic with 95% CIs. Improvements in prognostic classification were assessed using the net reclassification index (NRI) and integrated discrimination improvement (IDI). Clinical utility was evaluated through decision curve analysis. Kaplan–Meier survival curves were generated for visual comparison of groups, and log-rank tests were used to compare survival distributions. A 2-tailed *P* < 0.05 was considered statistically significant. All statistical analyses were conducted using SPSS version 27 (IBM Corp) and R studio (R Foundation for Statistical Computing).

## Results

A total of 291 patients were studied, with a median follow-up of 3.0 years (Q1-Q3: 3.0-3.0 years) and a survival time range of 0.02 to 3.0 years. Baseline clinical and hemodynamic data for the 4 groups are listed in Tables 1 and 2. There was a significant association between TFR, height, weight, and body mass index (all *P* < 0.05, Pearsons’s correlation) as expected.

**Table 1:** Baseline Demographic Characteristics

	All (N = 291)	Group 1: Low V-Q Concordance (n = 104)	Group 2: Low V-Q Discordance (n = 43)	Group 3: Normal V-Q Concordance (n = 102)	Group 4: Normal V-Q Discordance (n = 42)	*P* Value
Age, y	84 ± 8	82 ± 7	83 ± 7	82 ± 7	82 ± 7	0.339
Height, cm	165 ± 10	167 ± 12	171 ± 10	166 ± 9	167 ± 9	**0.026**
Weight, kg	75 ± 16	76 ± 17	85 ± 14	74 ± 16	68 ± 18	**0.000**
BMI, kg/m^2^	25 ± 9	22 ± 9	25 ± 8	22 ± 10	19 ± 8	**0.028**
BSA, m^2^	1.8 ± 0.3	1.8 ± 0.3	1.8 ± 0.3	1.8 ± 0.4	1.7 ± 0.3	0.489
Race/ethnicity						
Non-Hispanic White	285 (98)	103 (99)	42 (98)	100 (98)	40 (95)	>0.05 all
Non-Hispanic Black	2 (1)	1 (1)	0 (0)	1 (2)	0 (0)	
Black	0 (0)	0 (0)	0 (0)	0 (0)	0 (0)	
Other	4 (1)	0 (0)	1 (2)	0 (0)	2 (5)	
Sex						
Male	188 (65)	67 (64)	30 (70)	63 (62)	28 (67)	**0.033**
Female	103 (35)	37 (36)	13 (30)	39 (38)	14 (33)	
Laboratory results						
Creatinine, μmol/L	107 ± 57	136 ± 98	107 ± 41	112 ± 99	116 ± 94	0.280
eGFR, mL/min/1.73 m^2^	53 ± 24	46 ± 26	58 ± 22	61 ± 23	52 ± 22	**0.033**
Cholesterol, mmol/L						
Total	3.9 ± 1.1	3.8 ± 0.9	3.9 ± 1.1	4.1 ± 1.1	3.9 ± 1.3	>0.05 all
LDL	2.0 ± 0.9	1.9 ± 0.9	2.2 ± 0.7	1.9 ± 0.6	1.9 ± 0.6	
HDL	1.3 ± 0.5	1.1 ± 0.3	1.0 ± 0.5	1.6 ± 0.6	1.1 ± 0.1	
Ratio	1.7 ± 0.6	1.7 ± 0.8	2.2 ± 0.8	1.4 ± 0.6	1.7 ± 0.3	
Hb, g/dL	123 ± 17	124 ± 17	126 ± 17	121 ± 15	122 ± 20	0.733
Liver function tests, U/L						
ALT	18 [12–26]	20 [13–28]	17 [12–23]	17 [11–22]	17 [12–25]	>0.05 all
AST	25 [18–33]	27 [20–36]	24 [19–29]	25 [18–30]	22 [17–27]	
GGT	46 [22–68]	51 [26–72]	38 [20–54]	40 [21–60]	51 [25–77]	
ALP	80 [65–95]	85 [67–102]	75 [61–87]	73 [59–90]	83 [64–101]	
Platelet count, × 10^9^/L	197 ± 60	204 ± 61	195 ± 65	181 ± 62	216 ± 56	0.122
Comorbidities						
Anemia	124 (43)	51 (49)	16 (4)	48 (47)	9 (21)	0.132
Atrial fibrillation	24 (8)	9 (9)	4 (9)	8 (8)	3 (7)	0.460
Cancer	85 (29)	38 (37)	4 (9)	36 (35)	7 (17)	0.151
Carotid artery disease	74 (25)	28 (27)	12 (28)	23 (23)	11 (26)	0.168
Coronary artery disease	123 (42)	59 (57)	15 (35)	35 (34)	14 (33)	**0.033**
Chronic kidney disease	41 (14)	6 (6)	0 (0)	8 (8)	1 (2)	0.329
Chronic lung disease	76 (26)	28 (27)	15 (35)	26 (25)	17 (40)	0.511
Depression/anxiety	46 (15)	16 (15)	9 (21)	17 (17)	4 (10)	0.283
Diabetes	9 (3)	16 (15)	1 (2)	17 (17)	2 (5)	0.085
Dialysis dependent	8 (3)	1 (1)	1 (2)	2 (2)	0 (0)	0.655
Gout	21 (7)	4 (4)	0 (0)	2 (2)	1 (2)	0.820
Hypertension	183 (63)	78 (75)	28 (65)	67 (66)	28 (67)	0.460
Hypothyroidism	29 (10)	2 (2)	0 (0)	1 (1)	3 (7)	0.447
Liver cirrhosis	3 (1)	1 (1)	1 (2)	0 (0)	1 (2)	0.811
Metabolic syndrome	8 (3)	4 (4)	3 (7)	1 (1)	0 (0)	0.413
Smoker	79 (27)	28 (27)	15 (35)	22 (22)	14 (33)	0.208
Medications						
No. of antihypertensives	1.3 [0.5-2.0]	1.2 [0.5-2.0]	1.4 [1.0-2.0]	1.3 [0.5-2.0]	1.3 [0.5-2.0]	0.716
ACE inhibitors	142 (49)	48 (46)	21 (49)	50 (49)	23 (55)	0.732
Beta-blockers	70 (24)	35 (34)	7 (16)	27 (26)	8 (19)	0.271
CCB	58 (20)	15 (14)	11 (26)	20 (20)	12 (29)	0.418
Diuretics	102 (35)	38 (37)	15 (35)	36 (35)	13 (31)	0.796

Values are mean ± SD or n (%) or [Q1-Q3]. **Bold** denotes statistically significant.

ACE = angiotensin-converting enzyme; ALP = alkaline phosphatase; ALT = alanine transaminase; AST = aspartate aminotransferase; BMI = body mass index; BSA = body surface area; CCB = calcium channel blockers; eGFR = estimated glomerular filtration rate; GGT = gamma-glutamyl transferase; Hb = hemoglobin; HDL = high-density lipoprotein; LDL = low-density lipoprotein; Q = flow; V = volume.

**Table 2:** Hemodynamic Analysis

Baseline Hemodynamics and TTE Characteristics	All (N = 291)	Group 1: Low V-Q Concordance (n = 104)	Group 2: Low V-Q Discordance (n = 43)	Group 3: Normal V-Q Concordance (n = 102)	Group 4: Normal VQ Discordance (n = 42)	*P* Value
Blood pressure						
SBP, mm Hg	132 ± 26	127 ± 27	140 ± 28	133 ± 24	138 ± 25	**0.024**
DBP, mm Hg	64 ± 14	66 ± 15	59 ± 14	64 ± 14	63 ± 13	0.681
MAP, mm Hg	89 ± 17	88 ± 17	90 ± 22	89 ± 16	90 ± 12	0.879
PP, mm Hg	69 ± 24	62 ± 24	78 ± 23	69 ± 20	74 ± 27	**0.001**
Arterial compliance, mL/mm Hg	1.2 [1.0–1.6]	1.0 [0.9–1.2]	1.3 [1.1–1.6]	1.2 [1.0–1.4]	1.2 [1.0–1.5]	**0.000**
TTE left ventricle						
LVEF (%)	43 ± 17	34 ± 14	38 ± 13	55 ± 17	45 ± 17	**0.000**
Aortic valve						
Mean gradient, mm Hg	36 ± 15	31 ± 16	38 ± 16	40 ± 12	37 ± 15	**0.000**
Peak gradient, mm Hg	61 ± 23	52 ± 24	63 ± 24	68 ± 20	64 ± 24	**0.000**
Peak velocity, cm/s	382 ± 76	349 ± 83	386 ± 72	409 ± 59	397 ± 72	**0.000**
Flow rate, mL/s	219 ± 68	158 ± 27	269 ± 52	267 ± 47	181 ± 35	**0.000**
AVA, cm^2^	0.68 [0.54–0.82]	0.65 [0.50–0.77]	0.71 [0.57–0.85]	0.67 [0.55–0.79]	0.70 [0.58–0.84]	0.072
SVi, mL/m^2^	36 ± 12	27 ± 6	29 ± 4	47 ± 9	43 ± 10	**0.000**
Aortic CSA, mm^2^	820 ± 142	806 ± 113	787 ± 91	827 ± 158	872 ± 179	0.092
AS category						
Severe	117 (40)	22 (21)	23 (53)	55 (54)	17 (54)	**0.000**
Discordant severe	94 (32)	20 (19)	7 (16)	45 (44)	22 (44)	
LF-LG severe	66 (23)	53 (51)	13 (30)	0 (0)	0 (0)	
Paradoxical LF-LG severe	14 (5)	10 (10)	4 (9)	0 (0)	0 (0)	

Values are n (%) or mean ± SD or [Q1-Q3]. **Bold** denotes statistically significant.

AS = aortic stenosis; AVA = aortic valve area; CSA = cross-sectional area; DBP = diastolic blood pressure; LF-LG = low-flow, low-gradient; LVEF = left ventricular ejection fraction; MAP = mean arterial pressure; PP = pulse pressure; SBP = systolic blood pressure; SVi = stroke volume index; TTE = transthoracic echocardiogram; other abbreviations as in Table 1.

**Figure 2:**
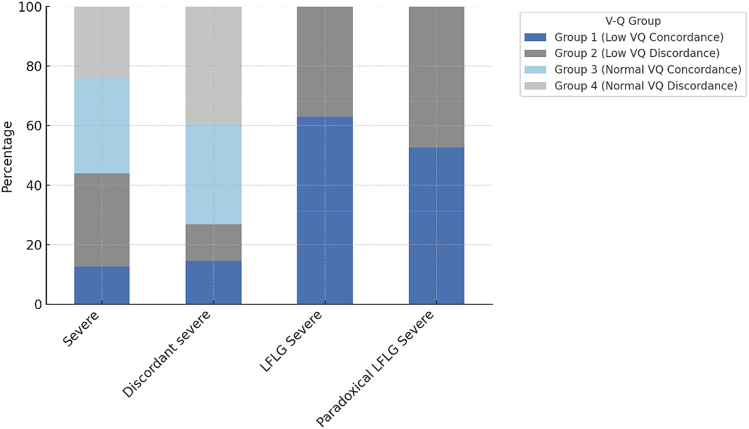
Distribution of V-Q Discordance Groups by AS Category Distribution of V-Q discordance phenotypes (groups 1-4) across AS categories. Bars represent the percentage distribution of each V-Q discordance group within AS categories, normalized to 100%. Group 1 (low V-Q concordance; blue) and group 3 (normal V-Q concordance; light blue) represent concordant phenotypes. Group 2 (low V-Q discordance; dark grey) and group 4 (normal V-Q discordance; light grey) represent discordant phenotypes. Although visual differences are evident across AS subtypes, the chi square test did not reach statistical significance (*P* = 0.109). LFLG = low-flow, low-gradient; Q = flow; V = volume; other abbreviation as in Figure 1.

**Figure 3:**
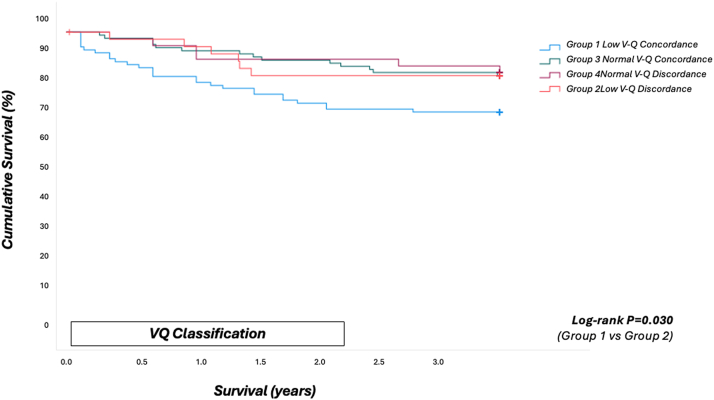
Kaplan-Meier Survival Curves Survival analysis of 291 study participants stratified by V-Q discordance group. Accompanying table shows the number of individuals at risk each year of follow-up stratified by group. A total of 43 deaths (15%) occurred during follow-up (median time to death: 1.0 years). Overall survival did not differ significantly across the four groups (log-rank *P* = 0.109). Three-year survival was comparable between the low V-Q discordance group (group 2) and the normal V-Q concordance group (group 3; log-rank *P* = 0.69). In contrast, 26 deaths occurred in participants with low V-Q concordance (group 1), compared with 6 in the low V-Q discordance group (group 2), corresponding to 3-year cumulative survival rates of 73.8% vs 86.0% (log-rank *P* = 0.030). Among participants with normal V-Q patterns, 17 deaths occurred in the concordant group (group 3) compared to 3 in the discordant group (group 4), with 3-year cumulative survival rates of 83.2% vs 92.9% (log-rank *P* = 0.135). Abbreviations as in Figure 2.

Low V-Q discordance (group 2) occurred in 29% (n = 43 of 47) of patients with an SVi ≤35 mL/m^2^ (vs 71% [n = 104 of 147] with low V-Q concordance [group 1]; *P* < 0.001) (Figure 2). Those with low V-Q discordance (group 2) were less likely to have comorbidities associated with degenerative stiffening of the aorta and/or arteriosclerosis. Specifically, hypertension was present in 65% (n = 28 of 43) vs 75% (n = 78 of 104) in group 1; coronary artery disease 35% (n = 15 of 43) vs 57% (n = 59 of 104); and diabetes 2% (n = 1 of 43) vs 15% (n = 16 of 104) (all *P* < 0.05) (Table 1, Figure 3). Diastolic BP was higher in group 2 vs group 1 (66 ± 15 mm Hg vs 59 ± 14 mm Hg; *P* = 0.018), whereas ascending aortic cross-sectional area (CSA) was lower (806 ± 113 mm^2^ vs 820 ± 142 mm^2^; *P* = 0.059), and arterial compliance higher (1.3 [Q1-Q3: 1.1-1.5] mL/mm Hg vs 1.0 [Q1-Q3: 0.8-1.2] mL/mm Hg; *P* = 0.001), despite similar SVi and LVEF measurements (Table 2, Figure 3) (both *P* > 0.05). Transvalvular hemodynamic indices were otherwise comparable between groups 1 and 2 (all *P* > 0.05) (Table 2).

Normal V-Q discordance (group 4) was observed in 29% (n = 42 of 144) of patients with an SVi >35 mL/m^2^, compared to 71% (n = 102 of 144) with normal V-Q concordance (group 3) (Figure 2). There was no association between normal V-Q discordance (group 4) and comorbidities known to be associated with degenerative stiffening of the aorta and/or arteriosclerosis (all *P* > 0.05) (Table 1). There was no significant increase in vascular afterload measures (including BP and arterial compliance) between those with normal V-Q discordance (group 4) and those without (all *P* > 0.05) (Table 2). Despite an increase in aortic CSA (827 ± 158 mm^2^ vs 872 ± 179 mm^2^; *P* = 0.001), AS hemodynamic indices were otherwise well matched between groups 3 and 4 (all *P* > 0.05) (Table 2).

### Survival analysis

All 291 study participants had follow-up clinical outcome data available 3-years post TAVR as per study inclusion criteria. Forty-three deaths (15%) occurred during follow-up (median time to death, 1.0 years). On binary logistic regression analysis, V-Q classification was prognostic of mortality (β = 0.408; SE = 0.164; 95% CI: 0.086-0.730; AIC: 23.79; *P* = 0.013), and superior to both SVi ≤35 mL/m^2^ (β = −0.615; SE = 0.311; 95% CI: −1.224 to –0.007; AIC: 29.59; *P* = 0.047) and TFR <210 mL/s classification (β = −0.274; SE = 0.303; 95% CI: –0.068 to 0.320; AIC: 32.80; *P* = 0.049).

**Figure 4:**
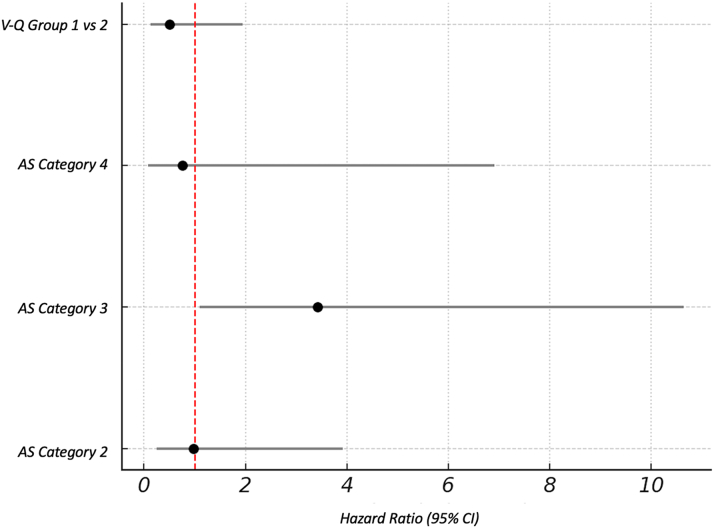
Adjusted HRs for All-Cause Mortality Comparing Groups 1 and 2 Forest plot showing results of a multivariable Cox proportional hazards regression model assessing the association between discordance phenotype and survival. Discordance group 2 was associated with a lower, though nonsignificant, hazard of mortality compared to group 1, with an adjusted HR of 0.50 (95% CI: 0.13-1.94). The model was adjusted for AS subtype, including AS category 2 (discordant severe AS), category 3 (LF-LG AS), and category 4 (paradoxical LF-LG AS), with category 1 (classical severe AS) as the reference. AS category 3 was significantly associated with increased mortality (HR: 3.42, 95% CI: 1.10-10.64). The vertical dashed line indicates the null effect (HR: 1.00). Error bars represent 95% CIs. Abbreviations as in Figure 2.

In a Cox regression model adjusted for age, sex, LVEF, and AVA, patients in group 2 (low V-Q discordance) had a lower, although nonsignificant, hazard of mortality compared to group 1 (low-volume/low-flow concordance) (HR: 0.80; 95% CI: 0.16-4.09). Among all pairwise comparisons, a statistically significant difference in survival was observed between groups 1 and 2 (*P* = 0.023), with group 2 demonstrating superior survival. After adjusting only for AS category, group 2 continued to show a numerically lower hazard of mortality compared to group 1 (HR: 0.50; 95% CI: 0.13-1.94). Additionally, patients classified as low-flow, low-gradient AS had significantly higher mortality risk compared to true severe AS (HR: 3.42; 95% CI: 1.10-10.64) (Figure 4) as expected.

**Figure 5:**
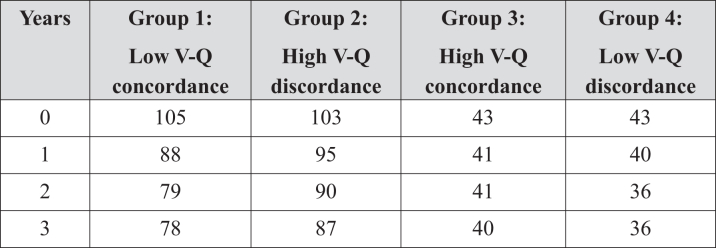
Number of Individuals at Risk Each Year of Follow-Up Stratified by Group Number of individuals at risk at each year of follow-up, stratified by volume–flow (V-Q) concordance and discordance groups. Group 1: Low V-Q concordance; Group 2: High V-Q discordance; Group 3: High V-Q concordance; Group 4: Low V-Q discordance. Values represent the number of participants remaining in each group at the start of each follow-up year (0-3 years).

Kaplan–Meier survival curves stratified by the 4 V-Q groups are shown in Figure 3. Interestingly, 3-year survival was similar between patients with low V-Q discordance (group 2) and those with normal V-Q concordance (group 3), with estimated survival rates of 86.0% (95% CI: 72.3%-95.1%) and 83.2% (95% CI: 74.6%-89.6%), respectively (log-rank *P =* 0.69). In contrast, 26 deaths occurred in patients with low V-Q concordance (group 1), compared with 6 in the low V-Q discordance group (group 2), corresponding to 3-year survival rates of 73.8% (95% CI: 64.3%-82.1%) vs 86.0% (95% CI: 72.3%-95.1%), respectively (log-rank *P =* 0.030). Among all those with normal V-Q patterns, 17 deaths were observed in the concordant group (group 3) compared to 3 in the discordant group (group 4), with 3-year survival estimates of 83.2% (95% CI: 74.6%-89.6%) vs 92.9% (95% CI: 75.5%-98.6%; log-rank *P =* 0.135) (Figure 5).

### Modelling

**Figure 6:**
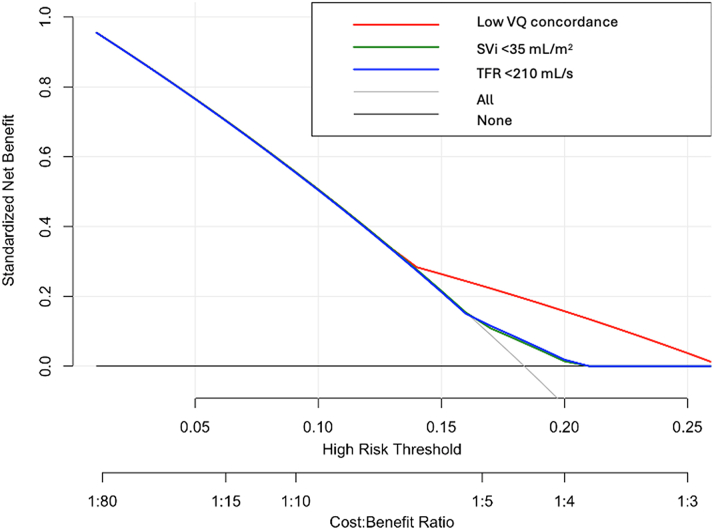
Decision Curve Analysis Decision curve analysis showing the standardized net benefit of using low V-Q concordance (group 1), SVi <35 mL/m^2^, and total flow reserve (TFR) <210 mL/min for predicting 3-year mortality after TAVR. Across a range of clinically relevant threshold probabilities (5%-25%), the low V-Q model (red) showed superior net benefit compared to conventional hemodynamic thresholds. The "all" and "none" curves represent strategies of treating all or no patients, respectively. These results support the added clinical utility of V-Q discordance in risk stratification beyond established flow-based metrics. TFR = transvalvular flow reserve; other abbreviations as in Figures 1 and 2.

The addition of V-Q discordance classification to a multivariable Cox model containing age, sex, LVEF, AVA, aortic valve velocity, and AS category led to a modest improvement in model performance. This included an increase in discrimination (C-statistic: 0.72 vs 0.68), improved model fit (likelihood ratio test: chi square = 4.11; *P* = 0.043), and a reduction in AIC (320.4 vs 322.5) (Table 3), as well as a significant improvement in reclassification (NRI = 0.24, *P* = 0.03; IDI = 0.06, *P* = 0.04). In contrast, traditional thresholds such as SVi ≤ 35 mL/m^2^ and TFR <210 mL/min demonstrated weaker prognostic performance. Neither was significantly associated with mortality in multivariable models (SVi HR: 1.26, *P* = 0.404; TFR HR: 1.29, *P* = 0.355), and both yielded lower C-statistics (0.531 and 0.535, respectively) than the V-Q discordance model (0.591). Reclassification analysis favored low V-Q concordance (group 1), with an NRI of +0.184 (95% CI: –0.02 to +0.48) vs SVi, and +0.116 vs TFR. Decision curve analysis further demonstrated superior clinical utility for the V-Q model across threshold probabilities of 5% to 25% (Table 3, Figure 6).

**Table 3:** Model Comparison Summary

Metric/Predictor	Base Model (No V-Q)	Full Model (With V-Q)	Group 1 (Low V-Q Concordance)	SVi <35 mL/m^2^	TFR <210 mL/s
C-statistic (overall)	0.68	0.72	0.591	0.531	0.535
C-statistic (low V-Q subgroup)			0.409	0.447	0.453
HR (Cox)			2.05 (1.20-3.51)	1.26 (0.73-2.16)	1.29 (0.75-2.22)
*P* value (Cox)			0.009	0.404	0.355
Log-likelihood	−154.24	−152.19			
AIC	322.48	320.37			
Likelihood ratio (chi square)		4.11			
*P* value (LRT)		0.043			
NRI vs SVi <35 mL/m^2^			Ref		+0.184 (–0.02 to +0.48)
NRI vs TFR <210 mL/s			Ref	+0.116	
DCA performance			Best	Lower	Lower

Hazard ratios are shown with 95% confidence intervals in parentheses. For NRI, values are reported as the point estimate followed by the 95% confidence interval in parentheses. All other values are reported as absolute estimates unless otherwise specified.

AIC = Akaike information criterion; DCA = decision curve analysis; LRT = likelihood ratio test; NRI = net reclassification improvement; Ref = reference; TFR = transaortic flow rate; other abbreviations as in Tables 1 and 2.

## Discussion

In this study, V-Q discordance classification emerged as a clinically meaningful predictor of survival in patients undergoing TAVR. Among patients with reduced SVi (≤35 mL/m^2^), those with preserved transvalvular flow rate (TFR ≥210 mL/s; group 2) had significantly improved 3-year survival compared to those with concordantly low SVi and TFR (group 1). This discordant phenotype also demonstrated superior prognostic value over SVi or TFR thresholds alone and was associated with a favorable hemodynamic profile, including lower vascular stiffness and improved arterial compliance. In contrast, V-Q discordance had no prognostic relevance among patients with preserved SVi (>35 mL/m^2^), highlighting its specific utility in the context of low-flow states (Central Illustration).

**Central Illustration:**
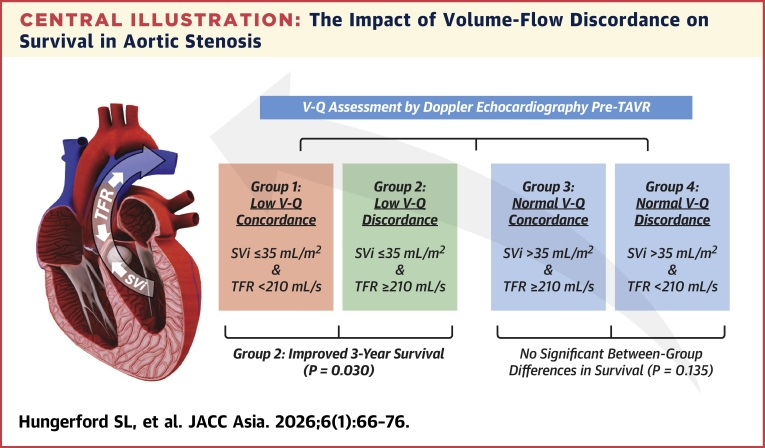
The Impact of Volume-Flow Discordance on Survival in Aortic Stenosis Patients were classified into 4 hemodynamic groups based on stroke volume index (SVi) and transvalvular flow rate (TFR). Group 1 (36%), classified as low V-Q concordance, had an SVi ≤35 mL/m^2^ and a TFR <210 mL/s. Group 2 (15%), defined as low V-Q discordance, had an SVi ≤35 mL/m^2^ but paradoxically a TFR ≥210 mL/s. Group 3 (35%), categorized as normal V-Q concordance, had an SVi >35 mL/m^2^ and a TFR ≥210 mL/s. Group 4 (14%), defined as normal V-Q discordance, had an SVi >35 mL/m^2^ but a TFR <210 mL/s. Among patients with an SVi ≤35 mL/m^2^, those with low V-Q discordance had significantly better 3-year survival (*P* = 0.030, log-rank), comparable to patients with an SVi >35 mL/m^2^. AS = aortic stenosis; Q = flow; SVi = indexed stroke volume; TAVR = transcatheter aortic valve replacement; TFR = transaortic flow rate; V = volume.

Approximately 14% (n = 20 of 144) of patients with a “normal” SVi (>35 mL/m^2^) had reduced TFR (<210 mL/s), whereas 15% (n = 22 of 147) with low SVi had preserved TFR—reaffirming that volume and flow are not interchangeable. Although the 210-mL/s TFR threshold has been widely adopted based on prior studies,^1^^,^^3^, ^4^, ^5^^,^^11^^,^^18^ Springhetti et al^7^ recently determined a higher cutoff of 218 mL/s in moderate AS. Supporting this, our data did not yield a data-driven optimal cutoff for TFR (best area under the curve [AUC]: 0.43; maximum Youden’s index: 0.05), underscoring the need for further validation and standardization of TFR thresholds.

V-Q discordance likely reflects the summative influence of both left ventricular contractility and vascular afterload. Prior studies have shown that TFR is sensitive not only to systolic function but also to vascular stiffness, independent of LVEF and SVi.^8^, ^9^, ^10^ In our study, patients with low V-Q discordance (group 2) were less likely to have comorbidities linked to arteriosclerosis and exhibited favourable vascular properties. This aligns with earlier findings showing that stress TFR is prognostically informative in low-flow, low-gradient AS.^11^^,^^18^

Several studies have emphasized the prognostic utility of TFR, both at rest and during stress, across the AS severity spectrum.^1^^,^^3^^,^^5^^,^^6^^,^^11^^,^^18^^,^^19^ Our findings build on prior work by demonstrating that V-Q discordance provides a modest yet statistically significant improvement in survival prediction, particularly among patients with low SVi. Although the absolute gains in model performance were limited — including a slight improvement in model fit, discrimination, and reclassification — these findings suggest that V-Q discordance captures hemodynamic nuances not fully accounted for by SVi or TFR alone.

Traditional AS severity assessment relies on anatomical and flow-dependent indices such as valve area, gradient, and SVi, which may be confounded by loading conditions. By incorporating TFR and assessing V-Q discordance, clinicians may better identify patients with discordant yet prognostically favourable physiology who would otherwise be misclassified. These findings support the integration of TFR and V-Q discordance into standard AS evaluation—particularly in those with low SVi — to refine risk stratification and guide treatment decisions.

### Study limitations

The retrospective design of this study presents inherent limitations, including missing clinical or echocardiographic data in 31% (n = 132 of 423) of eligible patients (Figure 1). Patients younger than 65 years of age were excluded to reduce heterogeneity in AS presentation and vascular physiology. While this approach limits confounding, it also excludes younger individuals—particularly women—who may exhibit distinct vascular profiles and in whom afterload may play a greater role in symptom development.^4^ The limited number of events (n = 43 of 291) introduces potential risk of model overfitting despite efforts to constrain covariate inclusion. As such, findings from the multivariable analysis should be interpreted with caution and validated in external cohorts. The 210-mL/s TFR threshold was initially derived in elderly TAVR cohorts but has since been applied to broader and more diverse populations, including younger individuals (eg, Namasivayam et al^1^), although its applicability across all age and sex subgroups remains to be confirmed. Future studies should explore whether age- and sex-specific vascular properties necessitate revised TFR cutoffs.^7^

### Future directions

Our findings confirm that V-Q discordance provides valuable prognostic insights in patients with low-flow states, even after TAVR. However, TFR appears to offer limited prognostic value in those with a baseline SVi >35 mL/m^2^, likely because left ventricular function plays a dominant role in this group. Nevertheless, these findings strengthen the case for incorporating the V-Q relationship as a simple, adjunctive baseline measure in patients with low-flow AS undergoing TAVR. Although a <210-mL/s threshold has been applied across a range of clinical contexts,^3^^,^^4^^,^^11^^,^^20^ future research should investigate whether alternative cutoffs—or indexing TFR to age, sex, or body surface area—may improve prognostic accuracy, particularly in patients younger than 65 years of age or those with preserved ejection fraction.

## Conclusions

Low V-Q discordance before TAVR is associated with improved 3-year survival in patients traditionally classified as having low-flow AS (SVi ≤35 mL/m^2^). Moreover, measuring the V-Q relationship provides incremental prognostic value beyond using SVi or TFR thresholds (≤35 mL/m^2^ and <210 mL/s, respectively) alone. Notably, there appears to be no additional prognostic benefit in assessing V-Q discordance in patients with “normal” flow at baseline (SVi >35 mL/m^2^). Overall, our findings highlight the value of V-Q discordance in providing additional hemodynamic insights into systemic circulation properties in patients with low-flow AS. Further research is needed to establish standardized reference ranges, which will enable a more comprehensive understanding of the potential for TFR in improving diagnosis, risk stratification, and prognostication in low-flow AS.

## Funding Support and Author Disclosures

Dr Hungerford has received grants from the National Heart Foundation of Australia Post-Doctoral and Vanguard awards. Dr Song has received grants from the Australian Government RTP. Dr Kapur has received personal fees from Abbott, Abiomed, Boston Scientific, Medtronic, MD Start, precardiac, and LivaNova. Dr Muller has received personal fees from Medtronic, Edwards Lifesciences, and Abbott Vascular. All other authors have reported that they have no relationships relevant to the contents of this paper to disclose.
